# On the Fundamentals of Reverse Ring Rolling: A Numerical Proof of Concept

**DOI:** 10.3390/ma17092055

**Published:** 2024-04-27

**Authors:** Ioannis S. Pressas, Spyros Papaefthymiou, Dimitrios E. Manolakos

**Affiliations:** 1 Laboratory of Manufacturing Technology, School of Mechanical Engineering, National Technical University of Athens, 9, Heroon Polytechniou Street, 15780 Athens, Greece; johnpressas@gmail.com (I.S.P.); manolako@central.ntua.gr (D.E.M.); 2 Laboratory of Physical Metallurgy, Division of Metallurgy and Materials, School of Mining & Metallurgical Engineering, National Technical University of Athens, 9, Heroon Polytechniou Street, 15780 Athens, Greece

**Keywords:** Reverse Ring Rolling, Ring Rolling, High-precision Manufacturing, Finite Element Analysis, Manufacturing Processes, Inconel 718 (IN718)

## Abstract

Ring Rolling is a near-net manufacturing process with some measurable dimensional inaccuracies in its products. This fact is exaggerated even more under the scope of high-precision manufacturing, where these imprecisions render such products unfitting for the strict dimensional requirements of high-precision applications (e.g., bearings, casings for turbojets, etc.). In order to remedy some of the dimensional inaccuracies of Ring Rolling, the novel approach of Reverse Ring Rolling is proposed and investigated in the current analysis. The conducted research was based on a numerical simulation of a flat Ring Rolling process, previously presented by the authors. Since the final dimensions of the ring from the authors’ previous work diverged from those initially expected, the simulation of a subsequent Reverse Ring Rolling process was performed to reach the target dimensions. The calculated deformational results revealed a great agreement in at least two of the three crucial dimensions. Additionally, the evaluation of the calculated stress, strain, temperature and load results revealed key aspects of the mechanisms that occur during the proposed process. Overall, it was concluded that Reverse Ring Rolling can effectively function as a corrective process, which can increase the dimensional accuracy of a seamless ring product with little additional post-processing and cost.

## 1. Introduction

Ring Rolling is a near-net manufacturing process, with the produced rings usually diverging from the intended dimensions. During a typical Ring Rolling process, the workpiece is rotated by the main roll, while the mandrel moves linearly towards the main roll to reduce the workpiece’s thickness, and increasing its radius [1]. At the same time, a set of conical rolls reduces the height of the ring at a different point of the ring’s periphery, thus leading to an overall complex deformation mechanism. This complex deformation mechanism combined with other phenomena manifesting during the process, such as defect formations [2] and instabilities [3], render the analytical calculation of the ring’s final geometry extremely difficult. As a result, an accurate prediction and control of the final dimensions of the workpiece cannot be efficiently achieved. Given that most Ring Rolling products are aimed at high-precision applications, for example bearing raceways (e.g., [4,5]), energy-producing machine parts (e.g., [6]), aircraft turbojet engine components (e.g., [7,8]) and spacecraft segments (e.g., [9,10]), the additional post-processing of its products is required to finalize them [11,12,13]. That being said, in the case in which the specific dimensions of the ring have overshot the allowable margins, the whole workpiece needs to scrapped, since there is no corrective action that can be carried out.

Because of the criticality of its products and in order to reduce the manufacturing time and cost, many researchers have investigated methods to further improve the precision and control of the Ring Rolling process. These methodologies facilitate a wide spectrum of tools available nowadays [14], and they are usually aimed at improving the control of the setup or to adaptively correcting the movement of the tools. The results of the proposed methodologies are subsequently validated either through finite element modelling and/or through experimental trials. An example of a real-time, closed-loop adaptive controller was presented by Zhang et al. [15]. The proposed controller facilitated fuzzy logic algorithms to adaptively control the rolls to reduce the eccentricity of the ring and increase its final circularity, while it was implemented in a corresponding finite element model. A similar work was, also, presented by Li et al., who developed an intelligent finite element model that calibrated the paths of the rolls, based on the temperature evolution on the workpiece [16]. In another work, Xie et al. correlated the mandrel feed rate in a radial Ring Rolling process with the geometric precision of the manufacturing rings and the overall stability of the process [17]. The aforementioned correlation was made from the comparison of the analytical expressions for eccentricity, circularity and vibration of the workpiece with the validated results from a coupled thermo-mechanical finite element model. The use of analytical expressions for the estimation of the ring’s dimensions and accumulated plastic strains was, also, proposed in [18]. A different approach was proposed in [19], in which the authors attempted to optimize some crucial Ring Rolling process parameters through a combination of numerical modelling, response surface methodology and statistical analysis, with the residual stress distribution on the workpiece being the objective response variable. Artificial intelligence has also been used as a Ring Rolling optimization tool [20]. In their work, Mirandola et al. developed an artificial neural network (ANN) to correlate several important parameters of the process with the energy consumption by the mandrel, while the necessary data for the ANN came from numerical simulations. Finally, Arthington, Havinga and Duncan presented a purely experimental control system for a precise Ring Rolling process, which optically recognized the geometry of the workpiece and calibrated the movement of its tools, based on the desired final product [21].

From all of the above, it is made clear that the main focus of most relevant analyses is to propose different optimization methods for Ring Rolling throughout the duration of the process. However, to the authors’ best knowledge, none of the presented analyses have attempted to correct the potentially created dimensional divergences after the end of the process. Such a corrective process is proposed and investigated in the current research. The main inspiration for the proposed process comes from the forming of ceramic pots. In pottery, it is a very common practice for a clay ring to be compressed radially while rotating, leading to a reduction in both of its radii. In case during this practice the clay ring’s thickness is kept roughly the same, the aforementioned radial reduction will result solely in an increase in the ring’s height. Given that at the end of most typical hot Ring Rolling processes the metallic ring remains at a high enough temperature that it can be subdued to further manufacturing, a similar radius reducing process, let it be called *Reverse Ring Rolling* hereafter, could be performed. Since the required equipment to investigate the feasibility of Reverse Ring Rolling experimentally was not available for the current analysis, a numerical simulation of the proposed process was the only alternative option. Furthermore, since the exact process parameters could not have been known from the beginning, as this is a newly proposed process, the investigation via a finite element model allowed for a safe way to test and determine the allowable margins of these parameters, without the risk of damaging the process equipment or hurting its user. It should be clarified that, from its conceptualization, Reverse Ring Rolling is primarily intended to correct relatively significant dimensional divergences of several millimeters and not common Ring Rolling defects like fishtails and/or bulges.

## 2. Methodology

In order to conduct the numerical simulation of the Reverse Ring Rolling process, a fully validated thermo-mechanical model of a typical hot Ring Rolling process was used as the foundation. More specifically, all the numerical, material and geometrical properties of the validated numerical model presented in [22] were implemented almost one-to-one in the simulations of the current analysis. Since the detailed presentation of all the simulation parameters would needlessly increase the length of the current paper, the reader is advised to refer to [22] for more information on the development of the aforementioned numerical model. It should only be mentioned that the material of the manufactured ring was IN718, while AISI H13 steel was the material of the tools. It is worth mentioning that the aforementioned Ring Rolling simulation was fully validated with experimental data found in [23].

### 2.1. Characteristics of Reverse Ring Rolling Process

**Figure 1:**
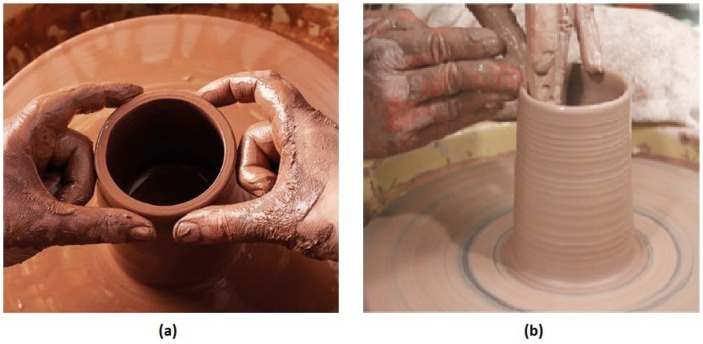
Applicable pottery techniques to Reverse Ring Rolling: (**a**) six-point “Collaring” (image taken from [24]) and (**b**) “Pulling” (image taken from [25]).

Before proceeding to the simulation of Reverse Ring Rolling, the basic mechanics of the process should be established. As a first step for this, the movement and positioning of a potter’s hands during the reduction of a pot’s radii were analyzed. From the plethora of techniques that potters perform in their art, two specific stood out as the most suitable for application in Reverse Ring Rolling. The corresponding techniques are presented in Figure 1.

The first technique (Figure 1a) is known as “collaring” or “choking” (the term “collaring” will be used hereafter) and involves the axisymmetrical squeezing of the clay ring to reduce both of its radii, while a small simultaneous increase in its height occurs. Usually, the clay ring’s thickness is maintained fairly constant during this process. The second technique (Figure 1b) is known as “pulling” and involves the wall thickness reduction of the clay ring, in order to increase its height. It should be mentioned that during the “pulling” technique, the inner radius of the clay ring remains roughly unchanged.

These two pottery techniques were attempted to be adapted for Reverse Ring Rolling. In order to do so, two separate numerical models were designed to validate the feasibility of the proposed process:

1. One for the adaptation of “collaring”, which would simulate the reduction of both ring radii up to a specific point.
2. Another for the adaptation of “pulling”, which would simulate the thickness reduction of the ring up to a target outer radius value.

In the case of the “collaring" Reverse Ring Rolling model, the six-point “collaring” technique was chosen as the basis for the model setup, in which the external peripheral pressure is applied through six equally spaced points to the workpiece. Given that the set of tools in the main rolling bite (main roll and mandrel) could be considered as one of the six points, the rest of the points would be covered by five separate support rolls being in contact with the ring. These support rolls would be spaced equally along the periphery of the workpiece, thus a $\theta_{spacing}$ = π/3 angular spacing from one point to the next was calculated. Regarding the movement of the tools, all rolls would move linearly and simultaneously towards the center of the ring. Additionally, a constant rotational velocity would be provided by the main roll, similarly to a typical Ring Rolling process. Because of the simultaneous movement of the tools, the ring wall thickness is expected to remain almost unchanged by the end of the process, while an increase in the ring’s height should be observed.

Regarding the “pulling” Reverse Ring Rolling model, the same tools to “collaring” would be used. Regarding the movement of the tools, the rotation of the ring would be caused by the main roll, in this case too, while the main roll and the five support rolls would move linearly towards the center of the ring. On the other hand, the mandrel would be restricted from any linear movement, although it would be still allowed to rotate freely. With the tools moving as described above, a stable thickness reduction in the ring and a simultaneous, measurable height increase should be performed, while potential process instabilities should be kept to a minimum. It is worth noting that the “pulling” process is intended for a total wall thickness reduction of only a few millimeters, as extending it for more would most probably lead to instabilities and, consequently, to major defects.

**Figure 2:**
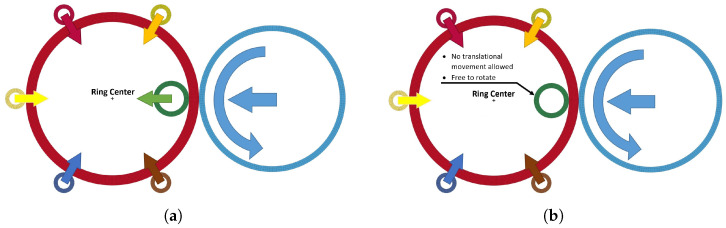
Applied movement on the rolls in each of the Reverse Ring Rolling process simulations: (**a**) “Collaring” model and (**b**) “Pulling” model.

A schematic representation of the applied movement on each of the tools in the proposed Reverse Ring Rolling processes is shown in Figure 2.

From all of the above, it is clear that for the successful adaptation of the pottery techniques to the Reverse Ring Rolling process, a Ring Rolling mill slightly different from a typical one is required. More specifically, multiple additional support rolls are required to perform these processes. Although uncommon, Ring Rolling mills with multiple, individually moving tools have already been presented in the literature. Most notably, Li, Guo and Wang mentioned the existence of an industrial four-support-roll mill, which used as a reference for their numerical simulations [26]. Additionally, in [27] a six-support-roll mill was used to perform incremental Ring Rolling on profiled rings. The Ring Rolling mill mentioned in [27] is referenced as a “flexible Ring Rolling” mill and has been used by the researchers of Cambridge University in multiple of their published works (e.g., [28,29,30], etc.).

Based on the aforementioned alternative Ring Rolling mills found in the literature, a suitable Reverse Ring Rolling mill should, at minimum, cover the following design points:

- Have more than two support rolls.
- Have a suitable mill table or rollers, which would support the workpiece, without hindering its free rotation.
- Allow for the fully controllable, independent movement (linear and rotational) of every individual tool.
- Have an appropriate number of measuring subsystems that would provide dimensional feedback of the manufactured workpiece.
- Have a central controller, which would allow for an a priori programming of the synchronous movement of the tools and/or their adaptive correction during the process.
- Have adequate cooling and heating systems to regulate the temperature of the workpiece and the tools.

It is worth noting that a Reverse Ring Rolling mill based on these points could also be used for traditional Ring Rolling; thus, any need for unnecessary breaks between the Ring Rolling, “collaring” and “pulling” processes would be eliminated, since only a proper tool velocity adjustment would be required to go from one process to the next. As a result, several metallurgical phenomena (e.g., recrystallization) that usually occur between the different processes of the production cycle, due to the movement and cooling of the material, would also be eliminated. Overall, even if a suitable, industrial-sized Reverse Ring Rolling mill does not exist at the moment, a proposed design based on the aforementioned points seems to be realistically plausible.

**Figure 3:**
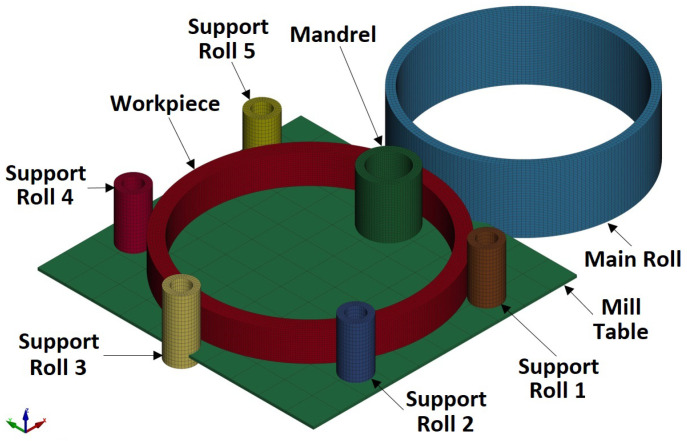
Ring Rolling setup used in the simulation of the “collaring” and “pulling” processes.

The Reverse Ring Rolling setup that was considered for both the “collaring” and “pulling” models in the current analysis is presented in Figure 3.

### 2.2. Reverse Ring Rolling Simulations

With the outlines of the analysis defined, some key aspects of the numerical models should be specified next. As mentioned before, the reference model of IN718 Ring Rolling presented in [22] was used as the basis for both models. This specific Ring Rolling model was considered as a good starting point for the current analysis, since it simulated numerous important thermo-mechanical phenomena of the process, such as multiple heat transfer mechanisms, temperature and strain rate dependent material properties and thermal expansion and shrinkage of the workpiece, among others. However, some alterations and adjustments had to be made, in order to properly simulate the proposed forming processes. These differences are discussed below separately for each of the “collaring” (Section 2.2.1) and “pulling” (Section 2.2.2) models. It is worth noting that the main goal of the Reverse Ring Rolling models is to correct the outer and inner radii of the manufacturing ring at the end of the simulation presented in [22], since its final dimensions diverged from those initially set as target dimensions (*R_f,target_* × *r_f,target_* × *H_f,target_* = 450 mm × 400 mm × 115 mm).

#### 2.2.1. “Collaring” Model Setup

As a first step, the initial geometry of the ring in the “collaring” model had to be determined. Seeing that Reverse Ring Rolling is proposed as a post-process performed right after the Ring Rolling process, the final geometry of the workpiece from [22] was input as the initial geometry in the “collaring” model. In short, the workpiece for the current analysis had an initial outer radius of *R*_0,*col*_ = 461.03 mm, an initial inner radius of *r*_0,*col*_ = 408.16 mm and an initial height of *H*_0,*col*_ = 116.1 mm. For reasons of simplicity, the cross-section of the workpiece was considered as a rectangle, as the inclusion of any fishtail and bulge defects would add an unnecessary level of complexity to the conducted analysis. The effects of these defects on Reverse Ring Rolling can be further investigated in a future analysis.

Regarding the geometry of the tools, all the rolls had the same dimensions as their respective counterparts presented in [22]. Additionally, a mill table was included in the current analysis. This table was simulated as a simple rectangular plate positioned right beneath the workpiece, with a side length of *a_table_* = 1000 mm (also a table thickness of 10 mm was considered, although this dimension was irrelevant). For the sake of simplicity, no roll slots were considered in the table’s geometry. Instead, each roll could move freely inside the table with no interaction between these bodies. The reason for the inclusion of a mill table was so that gravity could be added as an additional external load. Based on the mechanics of Reverse Ring Rolling, there is a great possibility that “ring climbing” could manifest during the process. The inclusion of gravity would help counter this instability to some extent, while also rendering the simulation more realistic. As a result, the addition of a mill table was necessary to support the workpiece and prevent it from moving out of the simulation boundaries. Finally, the conical rolls were omitted all together from the Reverse Ring Rolling models, since the ring height increase was an expected outcome of the process. However, one or more conical rolling bites could be included in a future study, in case height control or further vertical stabilization of the workpiece are required.

Regarding the movement of each tool, these would follow the outlines described in Section 2.1. More specifically, during the “collaring” model, all rolls would choke the workpiece until its inner radius would reach the target value of *r_f,target_* = 400 mm [23]. Given that *r*_0,*col*_ = 408.16 mm, a total radial movement of Δ*r_col_* = 8.16 mm would be performed by every roll, while the thickness of the workpiece should remain the same. All rolls would perform a linear movement from their starting position towards the ring’s center of rotation with the same linear velocity, while a slight delay would be considered from one roll to the next at the beginning of their movement, in order to compensate for their angular spacing. The linear and rotational velocities of the tools would be equal to $v_{mandrel}$ = 0.89 $\frac{\mathrm{mm}}{s}$ and $\omega_{MR}$ = 2.09 $\frac{\mathrm{rad}}{s}$, same to the corresponding velocities from the reference Ring Rolling model [22]. It is worth noting that the rotational velocity was applied only to the main roll, while the rest of the tools were able to rotate freely. The aforementioned velocity values were chosen as an initial approximation for the “collaring” model, since they were low enough not to introduce any significant dynamic phenomena to the process. In case any stabilization issues would be observed, however, lower velocity values would be tested.

Regarding the initial conditions, the boundary conditions and the external loads, the majority of these options were imported as-is from the reference model [22]. The only changes to those parameters, made in the Reverse Ring Rolling models, were the following:

- The initial temperature of the ring for the “collaring” model was set to $T_{ring,0,col}$ = 1313.15 K [22]. This value was considered to be the same for the entire ring.
- The boundary conditions applied on the mill table ensured a full constraint of all translation and rotation degrees-of-freedom (DOFs) for the corresponding body.
- Gravity was introduced in the “collaring” model. The gravitational load was considered only for the ring, since the rest of the bodies in the models were not allowed to move along the Z-axis (see Figure 3); thus, gravity was redundant for these bodies.
- Based on the imposed linear velocity of the tools and the target radii reductions, solution time had to be equal to $t_{col}$ = 9.17 s. However, since a minimum of 3 s had to be added so that the final dimensions would be normalized throughout the ring’s periphery, the total solution time was set to $t_{total,col}$ = 12.17 s.
- During the last 3 s of this simulation, when the dimensions of the ring would be normalized, all linear velocities of the tools were set to zero.

#### 2.2.2. “Pulling” Model Setup

**Figure 4:**
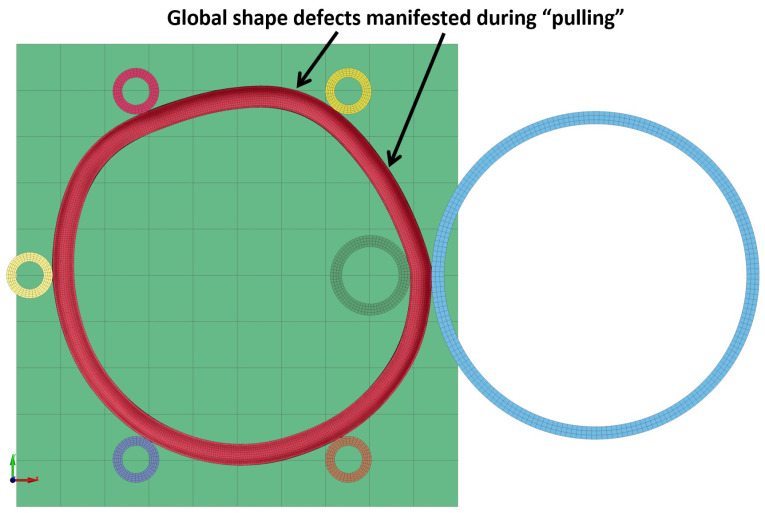
Global shape defects on the final product of a “pulling” process simulation, due to improper tool velocities.

After the inner radius of the ring would be reduced to its target value by the “collaring” process, the “pulling” process would be performed to correct the outer ring radius. In general, the “pulling” process is very similar to a finishing process in terms of the applied process parameters. Thus, a combination of a high rotational velocity and a relatively low linear velocity should be considered for the rolls. This fact was proven via preliminary trial models, in which different tool velocities (both linear and rotational) were tested. The trial models with tool velocities closer to those applied on the “collaring” model led to global shape defects on the final product. An example of a failed trial model with global shape defects is presented in Figure 4. From the preliminary trial models, a realistic process parameter set was determined.

Regarding the initial workpiece geometry for this model, the final state of the “collaring” model was used to define it, as well as, the initial tool positions. Other than that, the majority of process and model parameters were kept the same, since the general setups of the two processes were quite similar. Overall, the properties and parameters that were different in the “pulling” model, can be summed up to the following:

- The initial dimensions of the workpiece in the “pulling” model, namely its outer radius, its inner radius and its average height, were $R_{f,col}$ = 453.18 mm, $r_{f,col}$ = 400.01 mm and $H_{f,col}$ = 117.51 mm, respectively. Any pre-existing fishtail or bulge defects were not omitted from the current simulation, since they were an immediate result of the previous process.
- The initial temperature of the ring in the current analysis was re-evaluated, and it was slightly increased to $T_{ring,0,pull}$ = 1318.15 K, based on an approximation from the final temperature results of the “collaring” model, which was considered the same for the entire ring.
- The mandrel was not allowed to move linearly in the current process.
- The friction coefficients between the rolls and the workpiece were reduced to *FS/FD* = 0.2/0.1 for the main roll-to-ring contact and to *FS/FD* = 0.1/0.05 for the rest of the contacts (for the original friction coefficients please refer to [22]). Such friction coefficient values can be considered realistic, in cases of well-lubricated finishing processes, from which the quality of the product’s surface is a requirement.
- The rotational and linear velocity values considered in the current analysis were $\omega_{MR,pull}$ = $\frac{10\cdot \pi }{3}$$\frac{\mathrm{rad}}{s}$ and $v_{tools,pull}$ = 0.2 $\frac{\mathrm{mm}}{s}$, respectively.
- A slight delay was applied to the linear velocity of the support rolls. This delay was defined based on the respective angular position of each support roll and the angular velocity of the ring.
- Based on the final outer radius at the end of the “collaring” model ($R_{f,col}$ = 453.18 mm) and the target outer radius ($R_{f,target}$ = 450 mm), a total outer radius reduction of Δ$R_{pull}$ = 3.18 mm had to be performed during the “pulling” model. Given the linear tool velocity of $v_{tools,pull}$ = 0.2 $\frac{\mathrm{mm}}{s}$, a total simulation time of $t_{pull}$ = 15.9 s had to be applied. Since an additional Δ*t* = 3 s were at least required for the normalization of the ring’s dimensions, a total simulation time of $t_{total,pull}$ = 18.9 s was finally considered for the model.

It is worth noting that the stress, strain or temperature distributions were not transferred from the “collaring” to the “pulling” model, since the effects of the latter process needed to be solely evaluated. However, in an actual Reverse Ring Rolling process, the existence of residual stresses and/or non-uniform temperature distributions from “collaring” would potentially affect the outcome of an actual “pulling” process.

## 3. Results

With the conclusion of both simulations, their corresponding numerical results were inspected for errors or singularities, and subsequently they were evaluated. For reasons of clarity, each of the deformational, stress, strain, load and temperature results from each process are presented separately in the current chapter.

### 3.1. “Collaring” Model Results

An initial overview of the calculated results revealed that the process was completed as intended, with no visible defects or abnormalities. The rotation of the ring was continuous and unobstructed, while no vibrations were observed on the workpiece. Furthermore, both radii reductions were performed as scheduled (at a macro-scale level at least), while a slight increase in the workpiece’s height was also observed. In the following subsections, a more thorough presentation of some crucial results is made.

#### 3.1.1. Deformational Results (“Collaring” Model)

**Figure 5:**
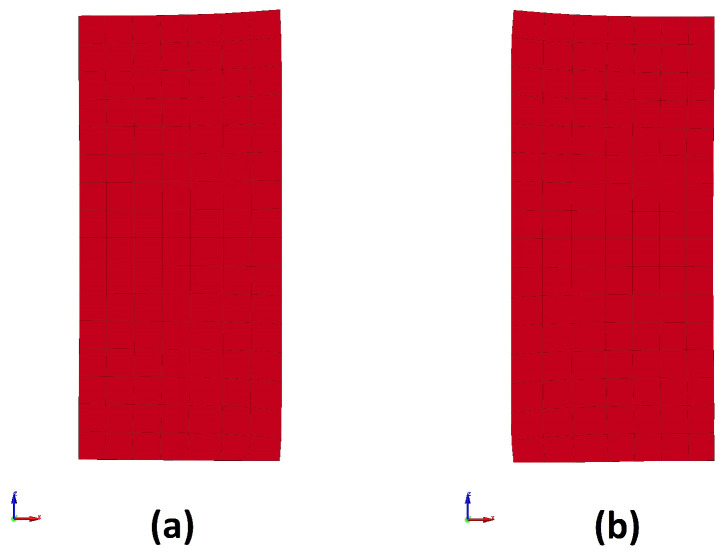
Cross-sections of the ring at the final time instance of the “collaring” model: (**a**) cross-section at the center of the main rolling bite and (**b**) cross-section in contact with a support roll.

Initially, cross-sections of the ring from the main rolling bite (between the mandrel and the main roll) and from the contact with a support roll (support roll 3 was chosen as reference, although similar results were observed in all support roll-to-ring cross-sections), at the final time instance of the simulation were extracted. These cross-sections are presented in Figure 5.

Observations over the cross-sections in Figure 5 revealed several interesting formations. More specifically, the cross-section at the center of the main rolling bite (Figure 5a) revealed the formation of fishtail defects on the right side of the cross-section, which was in contact with the main roll. The fishtail defect on the bottom right side of this cross-section was significantly smaller than that on the top right side, as a result of the mill table restricting its evolution. On the other hand, no fishtail defects were observed on the left side of the cross-section, which was in contact with the mandrel. Moreover, a very slight barreling could be observed on the right side of this cross-section. The aforementioned barreling can be attributed to the vertical movement of the material around the outer edges of the ring and to the friction with the main roll, which caused a small rotation of the formed fishtail defects and thus a barrel-like shape on the cross-section. An almost identical image was observed on the cross-section being in contact with the support roll (Figure 5b), in which the formed fishtail defects had little to no differences compared to the corresponding defects observed in Figure 5a, while the slight barreling was still present. Overall it can be concluded that during a “collaring” process, the reduction of both ring radii led to the increase in the average ring height mainly on the outer radii side. Additionally, the cross-section remained almost unaltered around the periphery of the workpiece.

**Figure 6:**
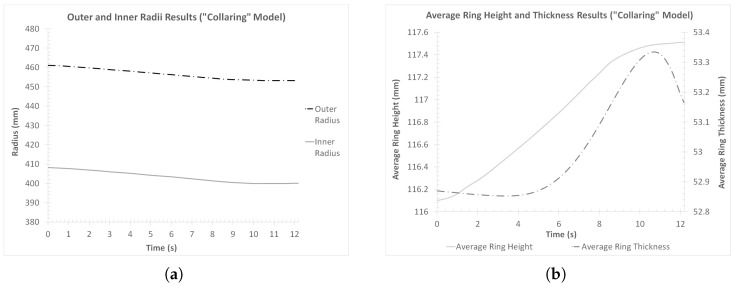
“Collaring” model deformational results: (**a**) outer and inner radii and (**b**) average ring height and thickness.

Afterward, the outer and inner radii results, as well as the average ring height and thickness results, were evaluated. For the estimation of each of the radii curves, data from twelve different diameters (uniformly spaced along the periphery of the ring) at the middle of the ring’s height were used. Furthermore, for the estimation of the average height curve, six different height measurements (uniformly spaced across each cross-section) from twenty-four cross-sections (uniformly spaced along the periphery of the ring) were taken. A similar method was also used for the estimation of the average thickness measurements, but with eight different thickness measurements (uniformly spaced across each cross-section) from twenty-four cross-sections (uniformly spaced along the periphery of the ring), in this case. The corresponding curves are presented in Figure 6a (outer and inner radii results) and Figure 6b (average height and thickness results), respectively.

Observations over Figure 6a revealed an overall steady reduction in both radii of the workpiece, throughout the main forming phase of the process (until $t_{col}$ = 9.17 s). The minimum outer and inner radii values were observed as expected at the end of the process. The corresponding final radius values were $R_{f,col}$ = 453.18 mm and $r_{f,col}$ = 400.01 mm, approximately. Since reaching the target value of the inner radius was the main scope of the current process, a comparison between $r_{f,col}$ and $r_{f,target}$ was initially performed. From this comparison, a difference of Δ$r_{col}$ = 0.0038% was observed, which can be considered negligible. In the case of the outer radius and compared to the corresponding target value $R_{f,target}$, a difference of Δ$R_{col}$ = 0.71% was observed. This difference, although relatively small, can be considered too large for a high-precision process. However, such differences can be affected by a more precise control of the rolls, or a smaller (and thus more controllable) linear velocity imposed on them. Additionally, other phenomena that affect the final dimensions of the workpiece, such as the thermo-elastic deformations of the tools or the ring’s shrinkage during its cooling down to room temperature, should also be taken into account and compensated for.

Regarding the average ring height (Figure 6b), an overall increase in this dimension was observed. During most of the main forming phase of the process, the increase rate of the ring’s height was fairly steady. Near the end of this phase and until the end of the process (after *t* = 8.5 s approximately), two lower increase rates could be discerned. For the first half of this duration (between *t* = 8.5 s and *t* = 10 s, approximately), the average ring height increased at almost half the rate it had until that point. After *t* = 10 s and until the end of the simulation, the height increase rate became even lower, and the average ring height remaining almost constant. The final average ring height value at the end of the process was equal to $H_{f,col}$ = 117.51 mm. Based on the final average ring height value of the reference model ($H_{f}$ = 116.1 mm [22]), the percentage average ring height increase was calculated equal to Δ*H* = 1.21%, which can be considered relatively small. It is worth noting that none of the individual height measurements used for the calculation of the average ring height were smaller than the target ring height value.

Regarding the average ring thickness (Figure 6b), a very slight decrease in the average ring thickness was observed initially (between *t* = 0 s and *t* = 4 s, approximately), which was subsequently followed by a sudden thickness increase, until *t* = 10 s, approximately. Then and until the end of the simulation, the average ring thickness steadily decreased until its final value of $f_{f,col}$ = 53.16 mm. In total, a percentage thickness difference of Δ*f* = 0.56% was recorded, which can be considered negligible.

In order to validate the stability of the “collaring” process, two additional dimensional results were reviewed, namely the inner and outer circularity of the workpiece and the ring center’s vertical displacement. For the calculation of circularity, *C*, of each peripheral surface, the corresponding equation (Equation (1)) found in the literature (e.g., [31,32,33]) was used.

(1) $$C=\frac{P^{2}}{4\cdot \pi \cdot A}=\frac{P^{2}}{4\cdot \pi^{2}\cdot r_{a}^{2}}\;\left(C=1,\;\mathrm{corresponds}\;\mathrm{to}\;a\;\mathrm{perfect}\;\mathrm{circle}\right)\;$$

where:

- *P* is the perimeter of the circle.
- *A* is the area of the circle.
- *r_a_* is the average radius of the circle.

**Figure 7:**
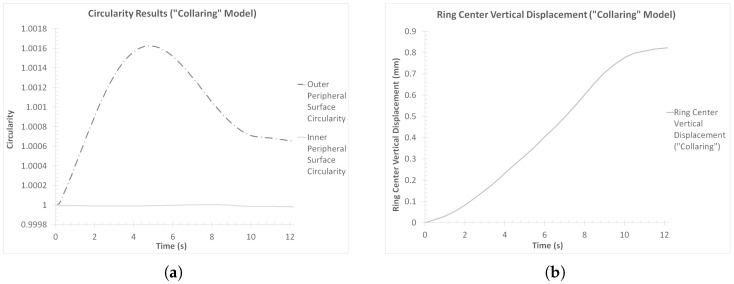
“Collaring” model additional deformational results: (**a**) outer and inner peripheral surface circularity results and (**b**) ring center vertical displacement.

For each of the peripheral surfaces of the workpiece, the outer and inner radii data previously used to calculate the average ring thickness curve (Figure 6b) were used to estimate the corresponding average radii. The corresponding perimeters were calculated by adding up the lengths of the linear element segment that corresponded to the same circles and then taking their average value. On the other hand, the ring center’s vertical displacement data were extracted directly from LS-PrePost v.4.9.6. The outer and inner circularity curves are presented in Figure 7a, while the vertical displacement curve is presented in Figure 7b.

Observations over the circularity results of the outer and inner peripheral ring surfaces (Figure 7a) revealed very small divergence from the optimum value of *C* = 1. More specifically, the average circularity of the outer peripheral surface at the end of the process was $C_{R,f,col}$ = 1.00065, while the same value for the inner peripheral surface was $C_{r,f,col}$ = 0.99998. Based on these results, it can be concluded that the effects of the “collaring” process on the circularity of the ring’s surfaces were minimal.

Finally, the vertical displacement of the ring’s center (Figure 7b) indicated a rather smooth overall process. More specifically, the corresponding result curve in Figure 7b showed a gradual elevation of the ring’s center over the duration of the process, with no oscillations observed. This behavior was expected, since the observed elevation of the ring’s center can be attributed to the height increase of the workpiece. On the other hand, if any oscillations were present in this curve, it would be indicative of an unstable process, possibly as a result of ring climbing.

#### 3.1.2. Stress and Strain Results (“Collaring” Model)

Regarding the stress distributions from the “collaring” model, a first review over the corresponding results revealed different behaviors between the two result sets (from the main rolling bite and from the contact with the support roll). More specifically, the Von Mises stress fringe plots from the main rolling bite cross-section were noticeably different from those of the cross-sections in contact with the support rolls. On the other hand, the effective strain fields were almost identical in every cross-section of the workpiece. Based on these observations, the Von Mises stress results from two separate cross-sections of the ring (one in the main rolling bite and another in contact with support roll 3) are discussed below, while the effective strain results from a single cross-section (that in the main rolling bite) are evaluated.

**Figure 8:**
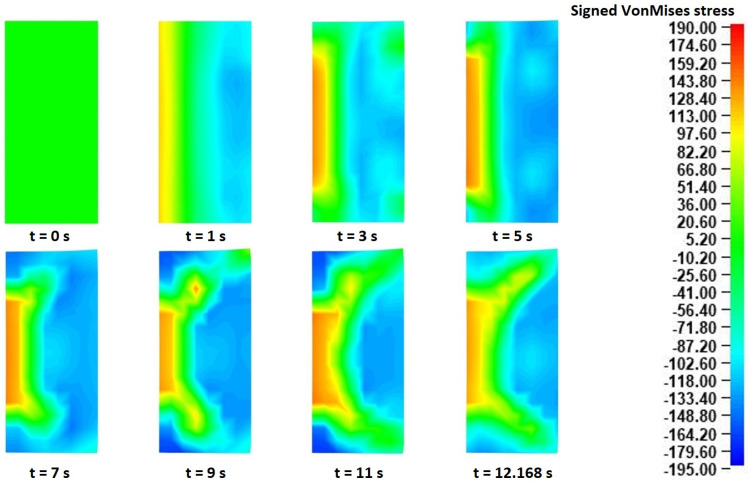
Signed Von Mises stress distributions at multiple time instances of the “collaring” Reverse Ring Rolling process simulation (Main Rolling Bite).

**Figure 9:**
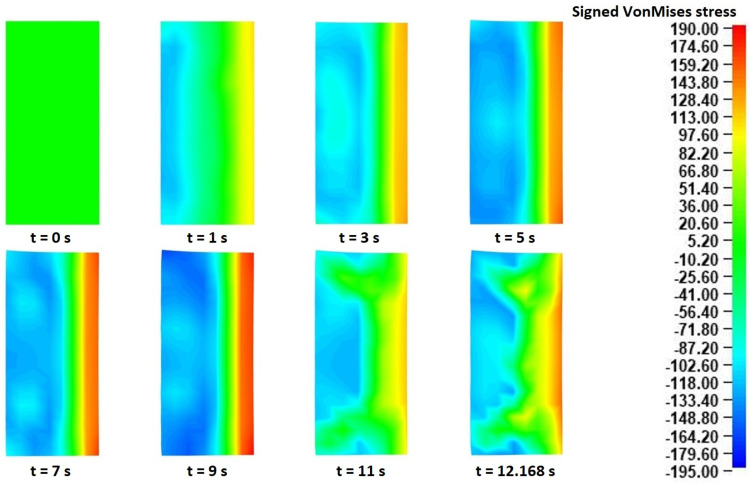
Signed Von Mises stress distributions at multiple time instances of the “collaring” Reverse Ring Rolling process simulation (cross-section in contact with Support Roll 3).

For this analysis, the signed Von Mises stress distributions at eight different time instances of the “collaring” model were exported. The signed equivalent Von Mises stresses have a similar calculation method to the equivalent Von Mises stresses, with the only difference being that the sign of the dominant stress values is maintained. In that way, the equivalent tensile and compressive stress results can be distinguished. In the current analysis, the use of signed Von Mises stresses helped to recognize the governing deformation mechanisms occurring during the “collaring” process. The corresponding signed Von Mises stress fields in each of the two aforementioned cross-sections are presented in Figure 8 (in the main rolling bite) and Figure 9 (in contact with support roll 3), respectively.

Observations over the signed Von Mises stress distributions of the cross-section in the main rolling bite (Figure 8) revealed an interesting stress evolution during the process. Before proceeding to the evaluation of these results, it should be noted that in each of the presented time instances in Figure 8 the main roll was located on the right side of the corresponding cross-sections and the mandrel was located on their left side. During the very early stages of the process (*t* = 0–1 s), a clear stress distribution could be observed in the analyzed cross-section, with a compressive stress field on the main roll side and a tensile stress field on the mandrel side. This specific distribution pattern can be attributed to a localized outward bending of the workpiece, caused by the action of the main roll. As the process proceeded (*t* = 3–5 s), some divergences from the initial stress distributions were observed, mainly around the edges of the workpiece. More specifically, some compressive stresses began building up around the inner edges, while the corresponding stress fields around the outer edges neutralized at first (*t* = 3 s) and then a slight compressive field began building up around them. From that point onwards and until the end of the process, an almost constant stress distribution was mostly observed, with the only exception being the stresses around the outer edges that gradually turned from purely compressive to almost zero. An overview of the stress field evolution helped clarify the deformational mechanism of the workpiece. More specifically, the initial compression from the main roll caused an outward bending of the ring, which, combined with the material continuity, led to a localized elongation of its inner peripheral surface. At the same time, a slight vertical arching of the cross-section was observed, which led to an early (*t* = 5 s) separation of the outer edges from the main roll, and subsequent pressure of the inner edges against the mandrel. After *t* = 7 s, the formation of fishtail defects along the outer edges was observed. The aforementioned defect formation led to the manifestation of compressive stresses around the bottom, outer edge being in contact with the mill table, while the corresponding upper outer edge stresses were closer to zero, due to a contact loss with the main roll. Finally, at the very end of the process the corresponding stress fields were noticeably reduced, as a result of the conclusion of the main forming actions and the normalization of the ring dimensions. From the entirety of the process, the maximum recorded (absolute) Von Mises stresses were observed near the end of the process (*t* = 11 s) around the inner edges of the ring, with the corresponding values being approximately 180–195 MPa.

Regarding the stress results observed in Figure 9, which were representative for all cross-sections being in contact with any of the support rolls, some significant differences were observed compared to the corresponding results presented in Figure 8. Firstly, in this case the corresponding tool was positioned on the left side of the presented cross-sections. For the majority of the process’s duration (*t* = 0–9 s), a localized outward bending of the ring was observed in this case, too. After *t* = 11 s, a notable change was observed in the corresponding stress fields, as the tensile stresses at the inner surface began reducing and the outer edge stress fields came closer to zero. The latter observation can be attributed to the fishtail defects bending slightly inwards and thus losing contact with the tool. Regarding the maximum recorded (absolute) stresses in Figure 9, these were observed near the end of the process (*t* = 9 s) around the outer edges of the ring, with the corresponding values being 170–190 MPa, approximately.

**Figure 10:**
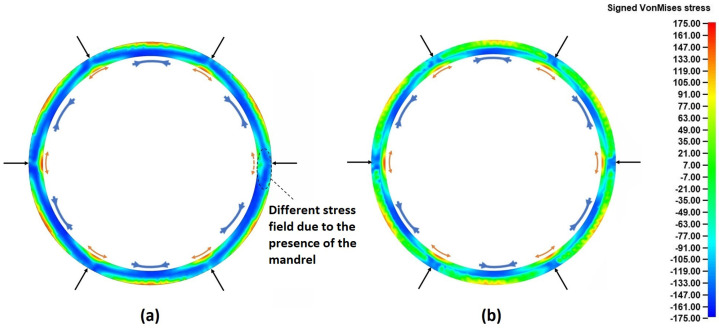
Signed equivalent Von Mises stress distributions at *t* = 5 s of the “collaring” Reverse Ring Rolling process simulation: (**a**) top view and (**b**) cross-section at the middle of the ring’s height. The black arrows indicate the radial compression from the rolls, the curved red double arrows indicate ring segments where tension is dominant, and the curved blue double arrows indicate ring segments where compression is dominant (along the inner peripheral surface).

The localized outward bending deformation mechanism observed in both Figure 8 and Figure 9 was of particular interest, and thus it was further analyzed. For this reason, the stress distributions from the entire workpiece at a representative time instance around the middle of the process (*t* = 5 s) were extracted. The aforementioned results are presented in Figure 10.

Observations over Figure 10 clarified and confirmed the deformation mechanism that was observed before. More specifically, from the stress fields presented in the top view of the ring (Figure 10a) tensile stresses appeared around the inner peripheral ring surface, of every roll contact point. The only exception to this was the main rolling bite, in which slightly compressive stress values were observed in the corresponding inner surface segment. However, combining these results to those previously observed in Figure 8 led to the conclusion that these compressive stresses were a result of the inner ring edges being compressed against the mandrel. This fact is further validated from the middle height cross-section of the ring (Figure 10b), where there was no contact with the mandrel and almost identical stress fields were observed around every roll contact point. Interestingly, in the arc segments of the workpiece located between two subsequent roll contact points, significant compressive stresses could be seen along the inner peripheral surface. Combined with the aforementioned observations, these compressive stresses indicate the formation of small hinge-like deformations, which were only restrained by the material’s own stiffness. Given that “collaring” is expected to be performed as a hot process and that the stiffness of most metals is significantly reduced in increased temperatures, these hinge-like deformations could potentially lead to global shape defects, in case of improper process parameters (mainly the linear and rotational velocities of the tools) are applied.

**Figure 11:**
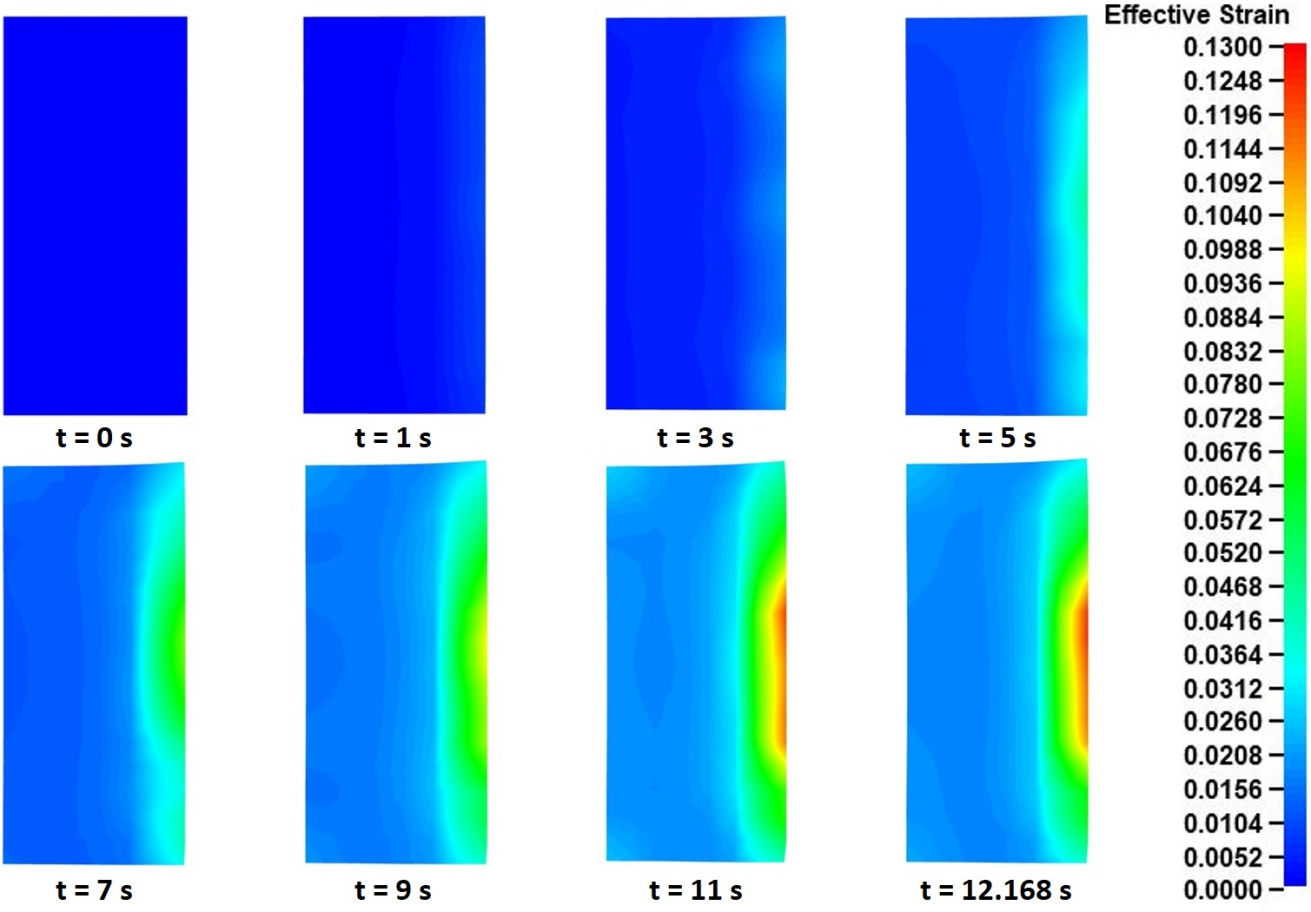
Effective strain distributions at multiple time instances of the “collaring” Reverse Ring Rolling process simulation (main rolling bite).

Afterward, the effective strain distributions were analyzed. For this analysis, the effective strain fields from a single cross-section needed to be extracted (the main rolling bite cross-section was chosen), since they were almost identical in every other cross-section of the workpiece. These effective strain distributions, at different time instances during the process, are presented in Figure 11.

Observations over Figure 11 revealed a rather expected strain evolution during the process. Since the main rolling bite cross-section was used for the current analysis, the positioning of the tools in Figure 11 was the same as that in Figure 8 (mandrel to the left, main roll to the right). Based on the mechanics of the “collaring” process, the main forming work is provided by the main roll and the support rolls to the outer peripheral surface of the workpiece. This phenomenon is clearly depicted in the effective strain results of Figure 11, which show a constantly increasing strain field concentrated around that surface. These strain fields increased steadily (both in intensity and volume) until approximately *t* = 11 s, when no further divergences were observed from that point and until the end of the simulation (*t* = 12.1685 s). The maximum recorded effective strains were observed after *t* = 11 s (and remained the same for the rest of the simulation) around the middle of the ring’s outer surface, with the corresponding values being approximately 0.12–0.13. Other than that, some relatively increased effective strain fields also appeared around the inner edges of the ring after *t* = 5 s, which also increased until the end of the process. The manifestation of these strain fields coincided with the forming and growth of fishtail defects, which caused the vertical arching of the cross-section and the subsequent pressing of the ring against the mandrel. The inner edge strain fields reached their maximum effective strains at *t* = 11 s, similarly to the outer peripheral surface strains. The maximum recorded inner edge effective strain values were approximately 0.03–0.04.

Overall, the evaluation of the stress and strain results helped to clarify some localized deformational phenomena and better comprehend the deformation mechanism that occurs during the “collaring” process. Since the observed results did not show any abnormalities or singularities, they can be considered as realistic and plausible.

#### 3.1.3. Load Results (“Collaring” Model)

**Figure 12:**
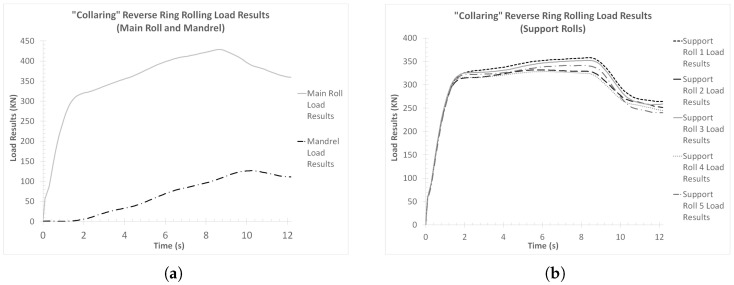
Load result curves from the “collaring” Reverse Ring Rolling process simulation: (**a**) main roll and mandrel loads and (**b**) support roll loads.

In the case of the load results from the “collaring” model, the corresponding curves were split into two different figures. In Figure 12a, the load reactions from the contact of the workpiece with the main roll and the mandrel are presented, while in Figure 12b the load reactions from the contact of the workpiece with each of the support rolls are presented.

Observations of Figure 12a revealed significant differences between the load curves from the main roll and the mandrel. More specifically, in the case of the main roll load curve, a rapid load increase was observed early on in the process (*t* = 0–1.5 s, approximately). From that point onwards and until *t* = 8.5 s, approximately, the main roll load continued to increase, although at a reduced rate compared to the beginning of the process. Additionally, at *t* = 8.5 s the maximum load value for the main roll was recorded at 430 KN, approximately. Lastly, from *t* = 8.5 s and until the end of the process, the main roll load decreased constantly, with a small change in rate after *t* = 10 s. The final main roll load value was approximately 360 KN.

On the other hand, the mandrel load curve was significantly lower compared to that of the main roll. In this case, the calculated load during the early phase of the process (*t* = 0–2 s, approximately) was negligible, indicating poor or no contact between the mandrel and the workpiece. After *t* = 2 s and until *t* = 10 s, approximately, a gradual increase at an almost constant rate was observed in the load, which ended at the corresponding curve’s peak value of approximately 130 KN. From that point and until the end of the simulation, the mandrel load decreased steadily, with the final value being 110 KN, approximately. From the comparison of both load curves presented in Figure 12a, the main roll load curve was measurably higher than the mandrel load curve throughout the process. In other respects, the two curves showed load increases and decreases at very similar rates, while the corresponding load changes mostly occurred at the same time windows during the process. The only differences to that were the first few seconds of the process, when no load was applied on the mandrel, and between *t* = 8.5 s and *t* = 10 s, when the main roll load decreased while the mandrel load increased. Especially, this last difference indicates that after the conclusion of the tools’ linear movement, the only active forming in the entire process was performed by the mandrel. This forming was mainly the normalization of the inner radius of the ring, throughout its perimeter.

Regarding the support roll load curves (Figure 12b), a first overview of these results revealed comparable load values amongst one another, as well as to those of the main roll load curve. In this case, all load curves were relatively close to one another, with the maximum percentage difference being approximately 12.5%. A more thorough analysis of these curves revealed a rapid initial load increase (from *t* = 0 until *t* = 1.5 s), which was almost identical to the corresponding segment of the main roll load curve. From that point onwards and until approximately *t* = 8.5 s, each load curve increased at a slightly different rate. Around *t* = 8.5 s, each curve reached its respective maximum load value, which ranged from 325 KN to 360 KN, approximately. After the conclusion of the main forming phase, two different load reduction phases could be observed in all curves, with a higher reduction rate at first (from *t* = 8.5 s until *t* = 10 s) and a lower after (from *t* = 10 s and until the end of the simulation). The final recorded load values of the curves in Figure 12b ranged from 240 KN to 265 KN, approximately. From the comparison of these load curves amongst one another, it can be deduced that the support rolls closer to the main rolling bite and the one directly opposite of the main roll (support rolls 1, 3 and 5) withstood greater loads compared to the other two support rolls (support rolls 2 and 4). Especially in the case of support roll 1 (also see Figure 3), an additional load percentage needed to be counterbalanced, since the dynamic component from the momentum of the ring applied some additional force to the aforementioned tool, as the latter exited the main rolling bite. It is worth noting that in case the rotational velocity had an opposite direction (counterclockwise instead of clockwise), the highest load curve would most probably correspond to the ring’s interaction with support roll 5.

From the correlation between all load results (Figure 12a,b) and the corresponding deformational results (Figure 6a,b), several interesting points can be deduced. Most notably, a clear correlation between the support roll load results and the deformation results was observed, since the corresponding curves in Figure 12b and Figure 6a,b showed rate changes at the same time instances. Furthermore, the rate changes observed after the main forming phase, indicated two different post-forming phases. At first (*t* = 8.5–10 s), a relatively quick normalization of the outer ring radii occurred, which in turn caused a relatively large height increase (compared to the next post-forming phase). After *t* = 10 s, however, only minor deformations took place (e.g., the ironing of potential defects) since the major ring dimensions had already been normalized and thus the need for additional work (and subsequently load) from the corresponding tools was significantly reduced.

#### 3.1.4. Temperature Results (“Collaring” Model)

**Figure 13:**
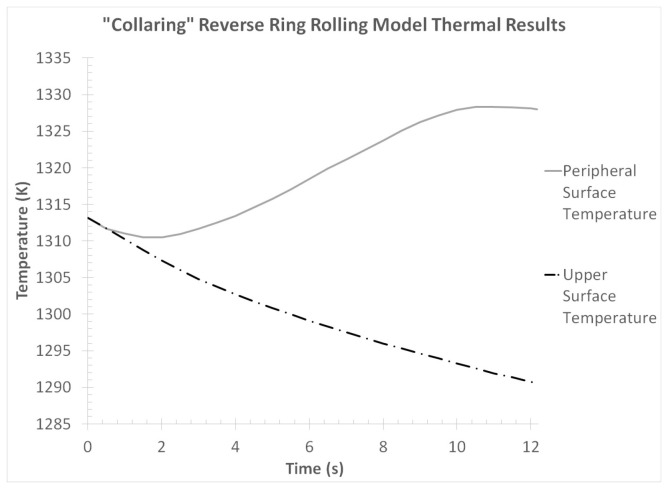
Temperature result curves from the “collaring” Reverse Ring Rolling process simulation.

Finally, the thermal results from the “collaring” model were evaluated. For this analysis, the average temperature curves from the ring’s outer peripheral and upper end surfaces were exported. The aforementioned temperature curves are presented in Figure 13.

Observations over the temperature curves in Figure 13 revealed some rather expected behaviors. In the case of the outer peripheral temperature, a slight reduction was observed early in the process (*t* = 0–2 s), which could be attributed to the relatively small deformations occurring at that time and the relatively high thermal conductivity of the tools. However, after *t* = 2 s, a gradual temperature increase was observed as a result of the plastic work turned to heat, which lasted almost until the end of the process (a slight temperature drop was observed at the very end of the process). The temperature increase rate was not constant throughout the process, with noticeable changes observed at *t* = 8.5 s, *t* = 10 s and *t* = 11.5 s. More specifically, at *t* = 8.5 s the temperature increase rate was reduced approximately in half, then between *t* = 10 s and *t* = 11.5 s almost no temperature increase could be observed and after *t* = 11.5 s a slight temperature decrease was recorded. These temperature rate divergences coincided with the changes in the forming process that were previously discussed. The maximum outer peripheral temperature was observed at *t* = 11 s, with the corresponding value being approximately $T_{peripheral,max,col}$ = 1328.3 K. However, the final outer peripheral temperature value was slightly lower at $T_{peripheral,f,col}$ = 1328 K.

On the other hand, a constant temperature reduction was observed in the upper end curve. Although the temperature reduction rate was not linear, a relatively smooth temperature curve was recorded. This was a rather expected behavior, since no forming was performed directly on the upper end surface of the workpiece and thus a constant heat transfer from the ring to the environment was the only thermal phenomenon that took place. The maximum temperature recorded in the upper end curve was the initially input value of $T_{upper,max,col}$ = 1313.15 K, while the temperature value at the end of the process was approximately $T_{upper,f,col}$ = 1290.7 K.

**Figure 14:**
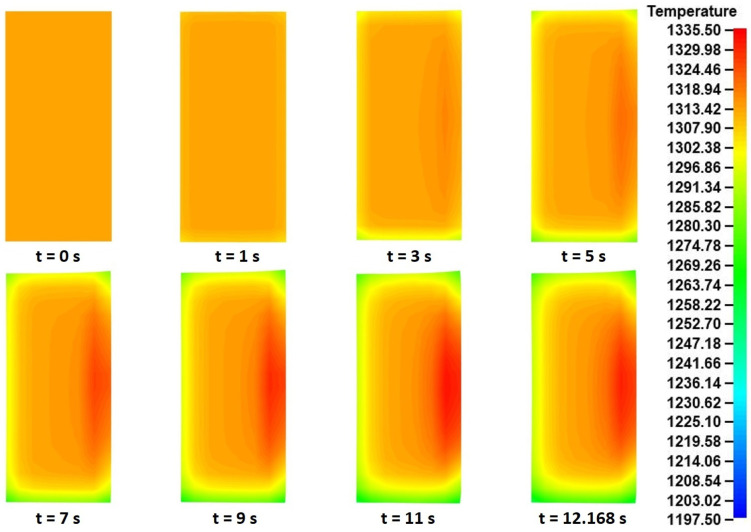
Temperature distributions at multiple time instances of the “collaring” Reverse Ring Rolling process simulation (main rolling bite).

Although very helpful, the peripheral and upper-end temperature curves provided little to no information about the temperature distributions inside the volume of the ring. For this reason, the temperature fields at various time instances of the process were extracted, in order to be further analyzed. A preliminary review of the temperature fields in various cross-sections of the workpiece and at the same time instances revealed that these fields were almost identical across the ring’s body. Thus, the corresponding temperature distributions from a single cross-section of the ring (the main rolling bite cross-section was chosen again) were extracted, and they are presented in Figure 14.

Observations over the thermal results in Figure 14 revealed a rather expected temperature evolution during the process. Before proceeding with the evaluation of these results, it should be noted that since the cross-section of the workpiece in the main rolling bite was used for the current analysis, the positioning of the tools was the same as the corresponding stress and strain field evaluations discussed above. During the early stages of the process (*t* = 0–3 s), a slight temperature decrease was observed in all surfaces of the workpiece, with the greatest temperature decrease occurring along the contact with the mill table. After *t* = 3 s and until *t* = 11 s, a similar temperature pattern was observed in all cross-sections. More specifically, the temperature of the bottom and top ring surfaces, as well as along the contact with the mandrel, was constantly decreasing, as a result of heat being transferred towards the tools and the environment. Interestingly, the temperature values recorded along the contact with the mandrel were always slightly higher compared to the corresponding distributions at the top and, especially, the bottom end surfaces, thus indicating that a percentage of plastic work was introduced by the mandrel. On the other hand, the temperature along the contact with the main roll kept increasing during the process, mainly due to the significant plastic work introduced by the aforementioned tool. This phenomenon could be observed until *t* = 11 s, while by the end of the process (*t* = 12.1685 s) a slight temperature decrease was observed in that area. The maximum recorded temperature from the entire process were observed along the contact with the main roll around *t* = 11 s, with the corresponding value being approximately $T_{max,col}$ = 1335.5 K.

### 3.2. “Pulling” Model Results

After the solution of the “pulling” model, the calculated results revealed a successful conclusion of the process. At first glance, the outer radius of the workpiece was reduced with a simultaneous increase in its height, while no abnormalities manifested. In the following subsections, a more detailed analysis of the calculated results is conducted.

#### 3.2.1. Deformational Results (“Pulling” Model)

**Figure 15:**
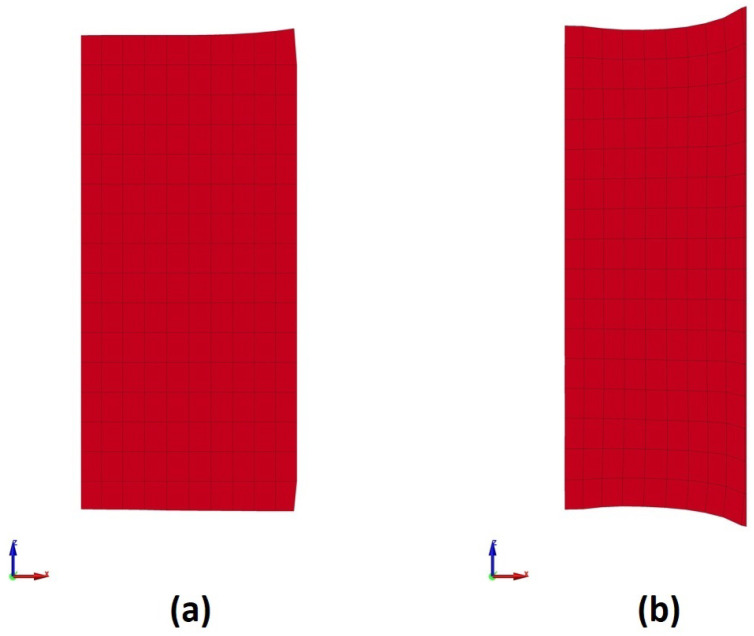
Cross-sections of the ring at different time instances of the “pulling” model: (**a**) initial time instance and (**b**) final time instance.

The deformational results from the “pulling” model were initially evaluated. As a first analysis, the cross-sections of the workpiece at the first and last time instance of the “pulling” model were reviewed in Figure 15. It is worth noting that the cross-sections in Figure 15 were almost identical in every cross-section of the ring, at their respective time instances.

Observations over the cross-sections in Figure 15 revealed a significant deformation of the workpiece during the “pulling” process. Regarding the orientation of the cross-sections in Figure 15, the outer peripheral surface was on their respective right side and the inner peripheral surface was on their left side. Most notably and apart from the thickness reduction, which was the main goal of the current process, large fishtail defects were observed at the outer edges, while smaller fishtail defects were formed at the inner edges. These fishtail defects were rather expected, as the majority of forming was conducted by the main roll and the support rolls. On the other hand, the formation of the inner fishtail defects implied that the workpiece was pressed at a measurable degree against the mandrel. It should be noted that the peripheral surfaces of the workpiece at the end of the “pulling” model had no arching, in this case.

**Figure 16:**
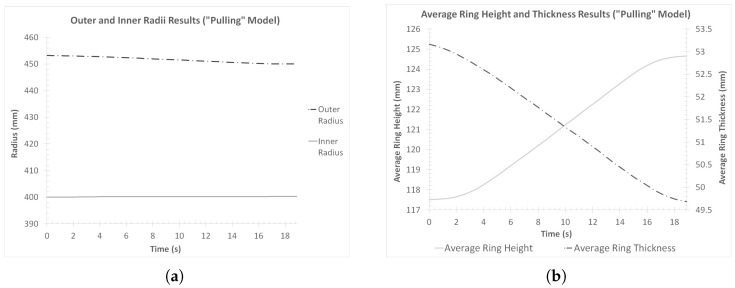
“Pulling” model deformational results: (**a**) outer and inner radii and (**b**) average ring height and thickness.

Subsequently, the dimension results were evaluated. The outer and inner radii curves and the average ring height and thickness curves are presented in Figure 16a,b, respectively.

Observations over the inner and outer radii, results presented in Figure 16a revealed a rather expected behavior. In the case of the outer radius results, a smooth (almost linear) reduction was observed from *t* = 0 s until *t* = 17.5 s, while little to no further deviations were recorded for the remainder of the process (*t* = 17.5–18.9 s). On the other hand, the inner radius results revealed a slight increase, which occurred at an almost linear rate during the process. The final outer radius value recorded at the last time instance was approximately $R_{f,pull}$ = 449.998 mm, while the final inner radius was equal to $r_{f,pull}$ = 400.31 mm, approximately. Compared to their respective target values, the inner radius was larger by a percentage of Δ$r_{pull}$ = 0.08%, while the outer radius was smaller by a percentage of Δ$R_{pull}$ = 0.0003%. Both of these percentage differences are small enough to be considered negligible, although the inner radius was increased from 0.0038% (at the end of the “collaring” process) to 0.08% greater than its respective target value.

Regarding the average ring height results, a rather expected behavior was observed in Figure 16b, too. During the early stages of the process (*t* = 0–3 s), a relatively slow increase in the average ring height was observed. From that point onwards and until the conclusion of the main forming phase (*t* = 3–15.9 s), the aforementioned dimension increased at an accelerated rate, as a result of the thickness reduction occurring at the same time. Finally, from *t* = 15.9 s and until the end of the process, another relatively slow increase was observed. The maximum average height was recorded at the end of the process, as expected, with the corresponding value being $H_{f,pull}$ = 124.66 mm. Based on this value, the corresponding percentage difference compared to the target height value was Δ$H_{pull}$ = 8.4%. However, both of these values can be considered as misleading, since the average ring height was heavily affected by the greatest ring height measured on the workpiece, which was located on the outer side fishtail defect. In order to have a more meaningful height result and given that the main goal of the ring height analysis was to ensure that there is enough material to grind off the ring so that the required target ring height can be reached, a measurement of the minimum ring height needed to be conducted. After reviewing various cross-sections of the final product, the minimum recorded height was found to be located closer to the inner peripheral surface, with its corresponding value being $H_{f,min,pull}$ = 121.22 mm. This height was Δ$H_{pull,min}$ = 5.41% greater than the corresponding target value, so additional grinding or machining post-processes could be performed to achieve the desired ring height.

On the other hand, the average ring thickness results (Figure 16b) followed the opposite pattern to the average height results, with an almost steady decreased observed throughout the process. The final average thickness value recorded was approximately $f_{f,pull}$ = 49.67 mm, with the percentage difference from the target value of $f_{target}$ = 50 mm being approximately Δ$f_{pull}$ = 0.62%, which is a rather small difference. It is worth noting, however, that since the final thickness was lower than the corresponding target value, a proper adjustment (reduction) of the total “pulling” process time should be applied to achieve the target thickness.

**Figure 17:**
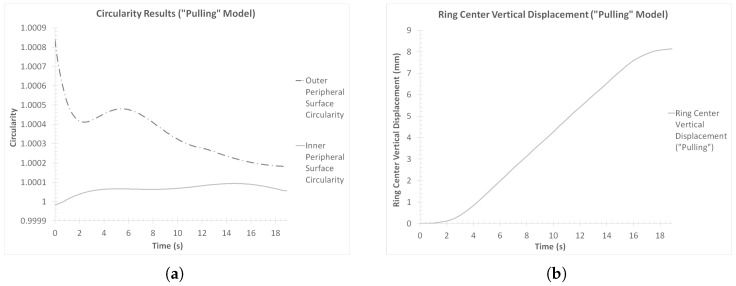
“Pulling” model additional deformational results: (**a**) outer and inner peripheral surface circularity results and (**b**) ring center vertical displacement.

Similarly to the “collaring” model, two additional dimensional results were reviewed, regarding the stability of the “pulling” model, namely the circularity of the two peripheral workpiece surfaces and the vertical displacement of the ring’s center. The corresponding curves are presented in Figure 17a,b, respectively.

Observations over the circularities of the outer and inner peripheral surfaces (Figure 17a) revealed generally good results. In the case of the outer peripheral surface, the calculated circularity seemed to decrease during the whole process, with a slight exception from *t* = 2 s to *t* = 5 s, when a small increase was observed. On the other hand, the inner surface circularity increased slightly during the process. The final circularity values were $C_{R,f,pull}$ = 1.00015 and $C_{r,f,pull}$ = 1.00005, respectively, which indicate an almost perfectly circular product (compared to the optimum circularity value of *C* = 1).

Furthermore, the ring center vertical displacement results (Figure 17b) also revealed a rather expected behavior. The corresponding curve increased smoothly throughout the process, which was anticipated as a result of the average height increasing during the process. The absence of oscillations indicates a steady process, with no ring climbing or other types of imbalances manifesting.

Overall, the deformational results proved the feasibility of the Reverse Ring Rolling process as a post-process of Ring Rolling, which can correct potential radial overshoots of significant sizes. Although the final dimensions from the conducted simulations were not exactly equal to the target dimensions (even if they were very close), this could be easily calibrated simply by adjusting the duration of each individual process.

#### 3.2.2. Stress and Strain Results (“Pulling” Model)

**Figure 18:**
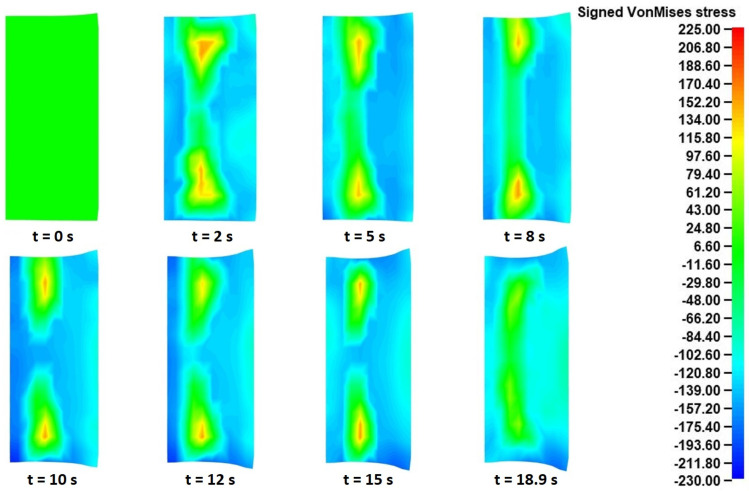
Signed Von Mises stress distributions at multiple time instances of the “pulling” Reverse Ring Rolling process simulation (main rolling bite).

**Figure 19:**
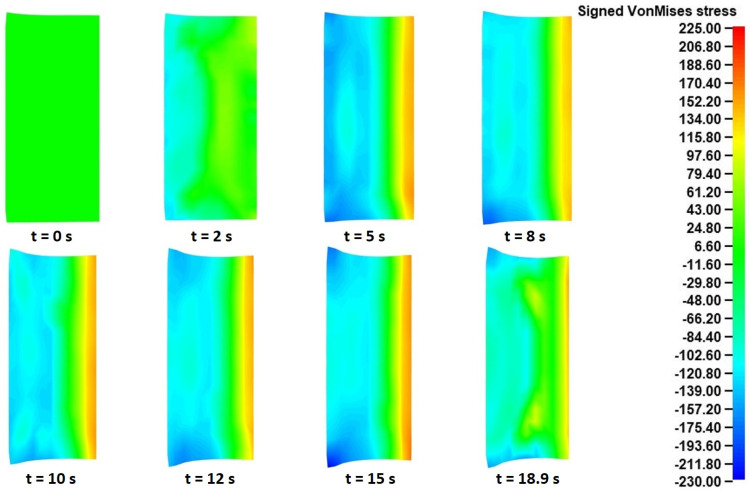
Signed Von Mises stress distributions at multiple time instances of the “pulling” Reverse Ring Rolling process simulation (cross-section in contact with support roll 3).

Following the deformational results, an analysis of the calculated stress and strain results from the “pulling” model was conducted. Initially, the signed Von Mises stress results at different time instances during the process were evaluated. Similarly to the “collaring” model, two different cross-sections of the ring were analyzed, namely one in the main rolling bite and another in contact with a support roll (support roll 3 was chosen in this case, too). The latter was compared with the corresponding cross-sections in contact with the rest of the support rolls and almost identical stress were observed among them. The aforementioned signed Von Mises stress results from the two cross-sections at different time instances during the process are presented in Figure 18 and Figure 19.

Observations over the signed Von Mises stress results in the main rolling bite (Figure 18) revealed some rather expected stress distributions. Before further analyzing the presented stress distributions in Figure 18, it should be reminded that the main roll was located on the right side of the depicted cross-sections, while the mandrel was located on their left side. In almost every time instance of Figure 18, two relatively high compressive stress fields were observed around the contacts with the main roll and the mandrel, with the latter having slightly greater (absolute) stress values. This fact can be attributed to the smaller contact area between the mandrel and the ring, compared to that between the main roll and the ring. On the other hand, a neutral to tensile stress field could be observed around the center of the corresponding cross-sections. This particular stress field was a result of the ring’s height increase, which produced these tensile stresses in areas where the vastly superior compressive stresses were not dominant or were completely absent. These two types of stress fields were observed throughout the process and around the same areas of the cross-section, with some relatively small deviations in shape and size. Finally, during the last stages of the process (*t* = 15–18.9 s), an additional compressive stress field was seen increasing around the bottom outer edge of the workpiece, as a result of the bottom fishtail defect interacting with the mill table. The maximum (absolute) stresses were observed around the inner edges of the workpiece and at the middle of the process’s duration (*t* = 10–12 s), with the corresponding (compressive) values being approximately 200–230 MPa.

In the case of the signed Von Mises stresses observed in the support roll contacting cross-sections (Figure 19), the corresponding distributions were also rather expected. Firstly, it should be reminded that the support roll in this case was located on the left side of the presented cross-sections. For the entirety of the “pulling” process, a relatively high compressive stress field was observed along the outer peripheral surface of the ring, as a result of the forming action of the contacting tool. On the contrary, a moderate tensile stress field could be observed along the inner peripheral surface during the process. The latter can be attributed to the same outward bending mechanism described in the corresponding results from the “collaring” model. These specific stress fields were observed almost unchanged throughout the process, with some minor deviations observed at the very first and very last instances of the simulation. The maximum (absolute) Von Mises stresses in this case were observed around the bottom outer edge of the ring at *t* = 15 s, with the corresponding (compressive) values being approximately 210–230 MPa.

**Figure 20:**
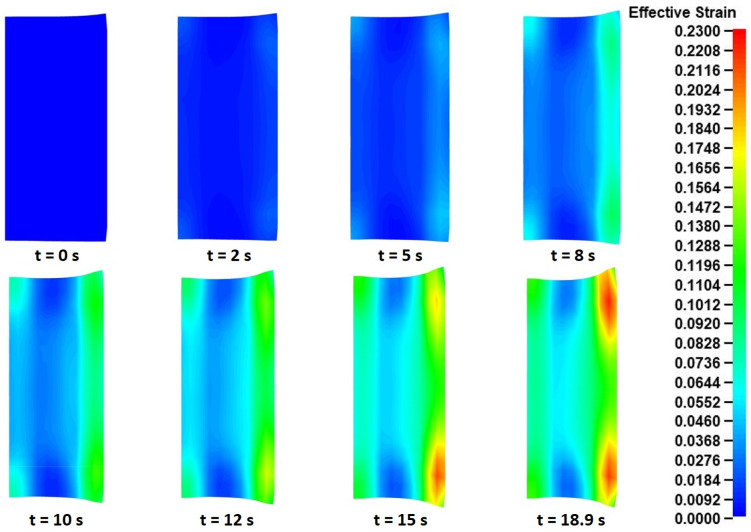
Effective strain distributions at multiple time instances of the “pulling” Reverse Ring Rolling process simulation (main rolling bite).

Finally, the calculated effective strain results from the “pulling” model were evaluated. Similarly to the “collaring” model, the strain results were almost identical along the perimeter of the ring; thus, only a single representative cross-section was analyzed (the main rolling bite cross-section was chosen). The corresponding effective strain distributions for multiple time instances during the process are presented in Figure 20.

Observations over the effective strain distributions in Figure 20 revealed a rather expected behavior. It is worth a reminder that in the main rolling bite cross-section that was chosen for this analysis, the outer peripheral surface is located on the right side of the presented instances, while the inner peripheral surface is located on their left side. During the “pulling” process, two distinct effective strain fields were mainly observed, one on each of the peripheral surfaces of the workpiece. These two effective strain fields increased in size and intensity over the process’s duration, with the strains on the outer surface size having slightly increased values compared to those on the inner surface side, at the same time instances. Interestingly, after *t* = 10 s and until *t* = 15 s, the calculated strains on the outer bottom edge of the ring began intensifying at an accelerated rate, compared to the strains at the upper outer edge. This phenomenon can be attributed to the compression of the outer bottom fishtail defect against the mill table, which contributed some additional strain to the existing strain field caused by the main roll’s compressive action. The maximum recorded effective strains from the entire simulation were observed around the outer edges of the ring at the end of the process, with their corresponding values being approximately 0.20–0.23.

Overall, from the stresses and strains analysis, some rather expected results were observed, based on the deformation mechanism taking place during the “pulling” process. The recorded stress and strain distributions had logical patterns, while no extreme values or abnormalities were observed.

#### 3.2.3. Load Results (“Pulling” Model)

**Figure 21:**
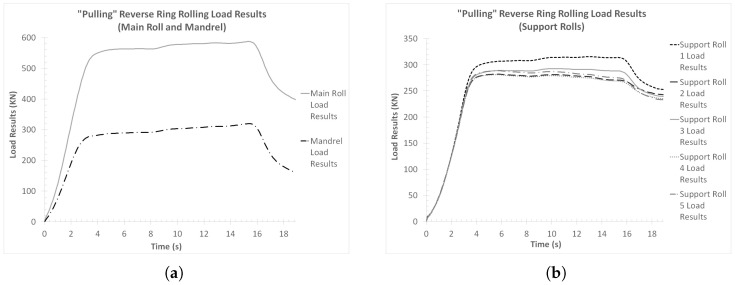
Load result curves from the “pulling” Reverse Ring Rolling process simulation: (**a**) main roll and mandrel loads and (**b**) support roll loads.

Next, the load results from the “pulling” model were analyzed. The corresponding load curves from the interaction of the workpiece with each of the tools were exported, and they were subsequently compared with one another. Similarly to the “collaring” model results, the curves of the main roll and mandrel loads were analyzed on a separate figure than the support roll load curves. The aforementioned curve comparisons are presented in Figure 21a,b.

Observations over Figure 21a revealed some rather expected load results. Both the main roll and the mandrel load curves had almost identical increase and decrease patterns, although their corresponding values were different. From the beginning of the process and until *t* = 3 s, a rapid load increase was observed in both tools. Afterward, the load increase rate decreased significantly, and the recorded load values increased only slightly until the end of the main forming phase (*t* = 15.9 s). On that point, the maximum load for each curve was recorded. After *t* = 15.9 s and until the end of the process, both curves gradually decreased. The maximum recorded load values for the main roll and the mandrel were 587 KN and 320 KN, respectively. In general, this behavior can be considered as logical, since the applied load by the main roll is counterbalanced both by the mandrel and by certain support rolls.

Regarding the load results from the ring’s reaction with each of the support rolls (Figure 21b), these were very close to the mandrel load curve from Figure 21a, both in terms of pattern and load values. All support roll load curves had a rapid initial load increase until *t* = 3 s, approximately, which was subsequently followed by a significant load deviation. Interestingly, only support roll 1 had an increasing load pattern after *t* = 3 s, while the rest of the curves either maintained a constant load value or their loads began decreasing. Finally, after *t* = 15.9 s, the loads in all curves decreased until the end of the process. The maximum recorded load appeared in support roll 1 curve at *t* = 15.9 s, with the corresponding value being approximately 315 KN. Generally, the observed load results followed rather expected patterns, while their respective values were within acceptable margins.

#### 3.2.4. Temperature Results (“Pulling” Model)

**Figure 22:**
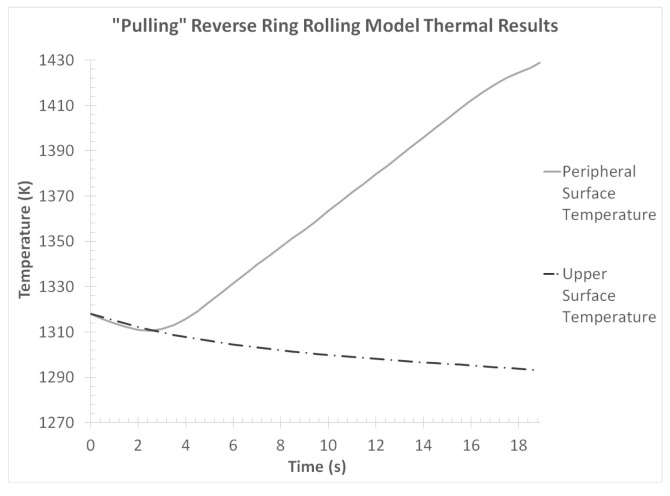
Temperature result curves from the “pulling” Reverse Ring Rolling process simulation.

Finally, the thermal results from the “pulling” model were evaluated. Similarly to the “collaring” model, the average temperature curves from the ring’s outer peripheral and upper end surfaces were analyzed. The corresponding temperature curves are presented in Figure 22.

Observations over the temperature curves in Figure 22 revealed some rather expected behaviors, which were very similar to the corresponding results from the “collaring” model. In this case, too, the peripheral surface temperature was initially reduced until *t* = 3 s, as a result of the tools’ high cooling rate combined with the relatively small deformation performed on the workpiece up to that point. However, after *t* = 3 s, a continuous and gradual temperature increase was observed on the peripheral surface, with the maximum temperature value at the end of the process being approximately $T_{peripheral,max,pull}$ = 1419 K. Interestingly after *t* = 15.9 s, a slight reduction could be observed in the temperature increase rate of this curve, since the main forming phase was concluded in that particular time. On the other hand, the upper surface temperature curve was constantly reducing throughout the process. This behavior was expected, since no direct forming was performed on the corresponding area of the workpiece and thus no plastic work, which would be turned to heat, was introduced there. The maximum temperature observed in the upper end curve was the initial value of $T_{upper,max,pull}$ = 1318.15 K, while the temperature at the end of the process was approximately $T_{upper,f,pull}$ = 1283 K. In general, the analyzed temperature curves had a rather expected behavior, with no particular points of concern.

**Figure 23:**
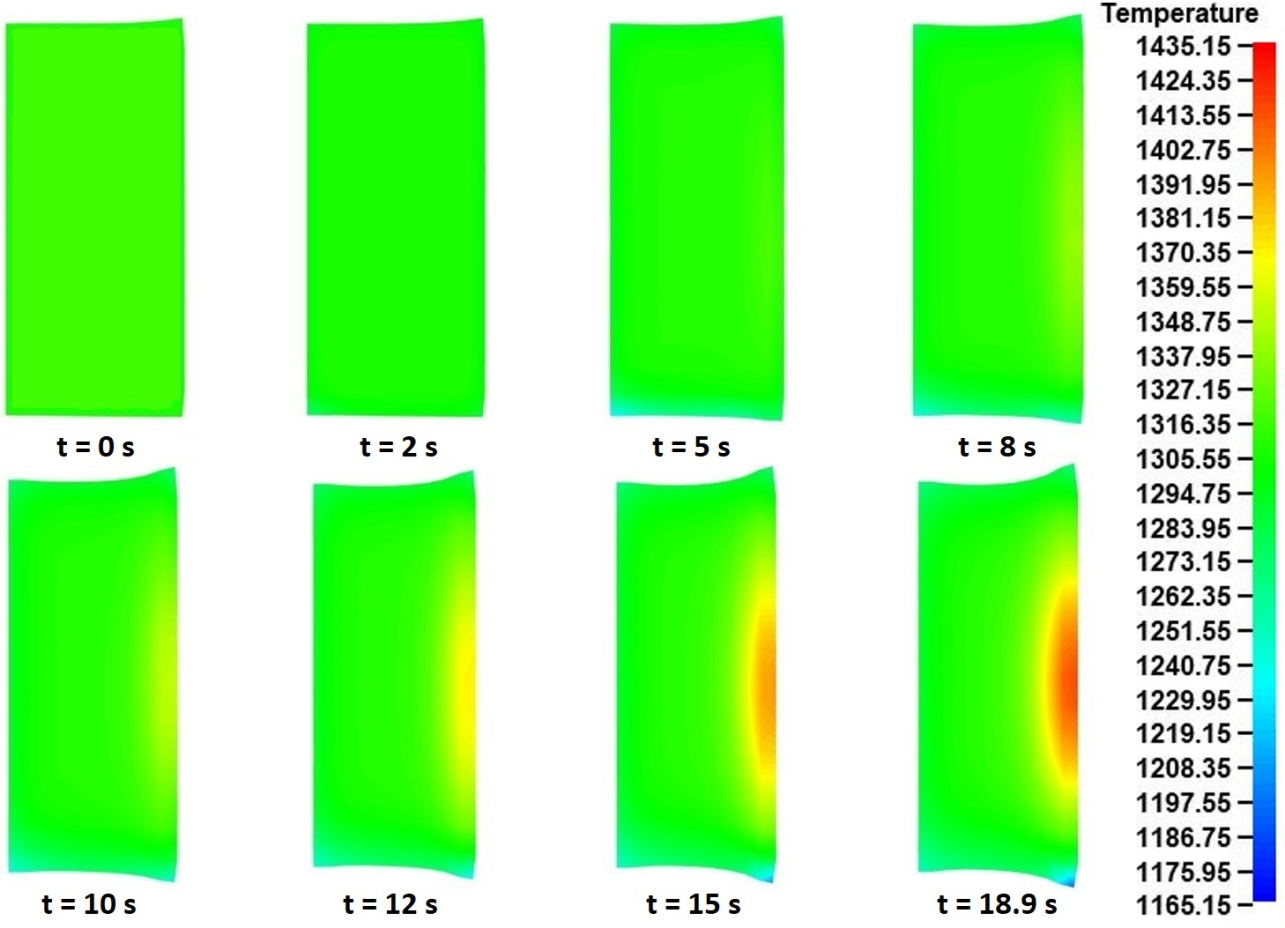
Temperature distributions at multiple time instances of the “pulling” Reverse Ring Rolling process simulation (main rolling bite).

Finally, in order to analyze the temperature distributions inside the volume of the ring, the corresponding fields at multiple time instances during the process were exported. From a quick review over different cross-sections of the workpiece, it was shown that the temperature fields were relatively uniform within the body of the ring. Thus, only a single cross-section of the ring (that inside the main rolling bite) was analyzed in the current analysis. The corresponding temperature fields are presented in Figure 23.

Observations over the temperature distributions in Figure 23 revealed some rather expected results. Prior to the analysis of the aforementioned results, it should be recalled that in Figure 23 the main roll is positioned on the right side of the presented instances, while the mandrel is on their left side. As with the “collaring” model, during the early stages of the process (*t* = 0–5 s) a temperature reduction could be observed mainly along the bottom surface of the ring, due to the ring’s contact with the mill table. From *t* = 5 s and until the end of the process, this temperature reduction concentrated on the outer bottom edge of the workpiece, while at the same time, a temperature increase was observed along the outer peripheral surface of the ring. The latter was a result of the plastic work introduced by the main roll’s compressive action. This temperature distribution gradually increased both in terms of size and of value over the duration of “pulling” process. Regarding the maximum and minimum temperatures in Figure 23 both were observed at the end of the process, with the corresponding values being $T_{max,pull}$ = 1435 K and $T_{min,pull}$ = 1165 K, respectively.

## 4. Summary

From the conducted simulations, the feasibility of a Reverse Ring Rolling process was verified. Both parts of this proposed process were simulated successfully, with no prohibitive issues or points of concern observed during the evaluation of their respective results.

More specifically, in the case of the “collaring” model a smooth reduction of both ring radii was performed, while the average height of the ring was increased. The majority of plastic deformations were performed across the outer peripheral surface of the workpiece, which led to the formation of one-sided fishtail defects. Moreover, from the evaluation of the equivalent stress, effective strain, load and temperature results some rather expected distributions were observed. Most notably, a deformation mechanism involving the creation of multiple hinge-like formations along the periphery of the ring was identified. Although this deformation mechanism caused no issues in the conducted simulation, it provided additional insight on potential global shape defect mechanisms that could manifest, in case improper process parameters are considered for the process. Overall, and compared to the target ring dimensions given in [23], an outer radius difference of Δ$R_{col}$ = 0.71%, an inner radius difference of much less than Δ$r_{col}$ = 0.01% and an average height increase of Δ$H_{col}$ = 1.21% were observed at the end of the “collaring” model. It is worth noting that although these percentages are relatively small, the corresponding differences in absolute values could still be considered as unacceptable for certain applications.

Contrary to “collaring”, the “pulling” model proved to be slightly more challenging. For this process, a number of preliminary trials had to be initially performed, in order to determine a set of viable process parameters that allowed for the successful conclusion of the process. When these process parameters were determined, the conducted “pulling” simulation proved to be stable and with very accurate results. The majority of the analyzed results were very similar to the corresponding results from the “collaring” model, thus no further comments will be made. Regarding the accuracy of the “pulling” process in terms of dimensional precision, the reduction of the outer ring radius was performed almost on spot, with its final value being Δ$R_{pull}$ = 0.0003% smaller than the target outer radius. At the same time, the average ring height had a percentage increase from Δ$H_{col}$ = 1.21% (at the end of the “collaring” process) to Δ$H_{pull}$ = 8.4%, with the minimum recorded height being greater by Δ$H_{min,pull}$ = 5.41% compared to the target height of $H_{target}$ = 115 mm. This fact ensures that enough material still existed on the ring, so that further grinding and/or machining processes could be performed on the end ring surfaces. It should be noted that because of the main roll pressing the workpiece against the mandrel, a slight inner radius increase was observed at the end of the “pulling” process, greater by Δ$r_{pull}$ = 0.08% than the corresponding target dimension. Although this difference could be considered significant in high-precision applications, it could be easily calibrated through some minor adjustments on the duration of each process. More specifically, a small undershoot of the inner radius could be performed during the “collaring” process, which would in turn balance the small overshoot observed in the same dimension, at the end of the “pulling” process. At the same time, the duration of the “pulling” process should be also slightly reduced to remedy the small undershoot observed in the outer radius, at the end of the “pulling” process. In general, the proper adjustment of the durations of these processes can (in principle at least) lead to the production of rings with two of their dimensions precisely manufactured.

Overall, the proposed process of Reverse Ring Rolling seems to be a highly promising addition to a typical Ring Rolling production plan. The main requirement of the process, which is a Ring Rolling mill with additional support rolls, has already been explored (to a certain degree) in the literature. On the other hands, the potential benefits, which mainly include the significant reduction or complete eradication of further radial post-processing for every product, can significantly reduce the duration and the cost of the total production. More specifically, the total duration of both processes in the simulated example was approximately $t_{total}$ = 31 s, and these processes could be performed right after Ring Rolling, with no intermediate breaks between them. Compared to a typical machining post-process, in which the workpiece has to be removed from the Ring Rolling mill, transferred to the machining setup, clamped and precisely offset and then machined in multiple cycles, the time difference between the two processes is marginally different. Additionally, removing a whole process from the production line of high-precision seamless rings will lead to a reduction in their final cost. However, even if the machining post-process cannot be entirely removed, reducing the number of machining cycles by a significant amount will also reduce the cost of the manufactured rings, as less damage will accumulate on the cutting tools and the machine, and thus fewer service cycles will be required.

Based on the proposed methodology of the current work, a future analysis should involve the experimental validation of Reverse Ring Rolling via an appropriate experimental setup, in order to solidly establish the viability of the proposed process. Other than that, the effects of several important process parameters (e.g., the tool linear and rotational velocities, the movement patterns of support rolls, the ring temperature and the number of support rolls) on Reverse Ring Rolling could be analyzed in-depth, in a future article. Finally, dimensional divergences caused by expected physical phenomena, such as the ring’s shrinkage due to its cooling to room temperature, could also be further analyzed and included in a future study of Reverse Ring Rolling.

## Data Availability

Data are contained within the article.
